# Reply: “Continuing the Chat: How Can We Improve the Performance of an Artificial Intelligence Chatbot in Answering Clinical Infectious Diseases Pharmacotherapy Questions?”

**DOI:** 10.1093/ofid/ofaf074

**Published:** 2025-02-06

**Authors:** Wesley D Kufel, Conan MacDougall, Elizabeth W Covington, Jason C Gallagher, Robert W Seabury, Jeffrey M Steele

**Affiliations:** School of Pharmacy and Pharmaceutical Sciences, State University of New York at Binghamton, Binghamton, New York, USA; State University of New York Upstate University Hospital, Syracuse, New York, USA; State University of New York Upstate Medical University, Syracuse, New York, USA; School of Pharmacy, University of California San Francisco, San Francisco, California, USA; Harrison College of Pharmacy, Auburn University, Auburn, Alabama, USA; School of Pharmacy, Temple University, Philadelphia, Pennsylvania, USA; State University of New York Upstate University Hospital, Syracuse, New York, USA; State University of New York Upstate Medical University, Syracuse, New York, USA; State University of New York Upstate University Hospital, Syracuse, New York, USA; State University of New York Upstate Medical University, Syracuse, New York, USA

To the  Editor—We thank Koh and colleagues for their correspondence and commend the authors for evaluating responses on our 100 developed clinical infectious diseases (ID) pharmacotherapy questions using the same rating scales and definitions [1]. A comparison of the ratings between our study and Koh and colleagues' evaluation can be found in Table 1 [1, 2]. The median correctness and safety ratings were both 7 and 8, respectively. However, Koh and colleagues found a higher median completeness rating of 6 as compared with 5 as described in our study. The most notable finding was the difference in the responses that were deemed useful. They rated the responses by GPT-4o to be 93% useful, as opposed to 41.8% as identified in our study.

**Table 1.** Rating Comparisons of ChatGPT Responses on 100 Clinical Infectious Diseases Pharmacotherapy Questions

	Median (IQR)			
	Correctness	Completeness	Safety	Useful, %
Kufel et al ^a^ [2]	7 (4–9)	5 (3–8)	8 (4–10)	41.8
Koh et al ^b^ [1]	7 (7–8)	6 (6–7)	8 (7–8)	93.0

^a^Ratings are based on GPT-3.5 responses.

^b^Ratings are based on GPT-4o responses.

The authors used multiple techniques to address some of the shortcomings described in our study and improve ChatGPT performance and response quality [1]. Unfortunately, GPT-4o was not available at the time of our study, and this version has demonstrated improved performance in biomedical knowledge domains and reductions in hallucination as compared with GPT-3.5 [3–5]. Furthermore, the versions of ChatGPT continue to evolve and be updated at a rapid pace, which makes timely evaluation of these versions logistically challenging [2]. Currently, the o1 model has been shown to have even better performance with its ability to reason through problems [6]. Their use of detailed prompts helped improve response quality and should be applied when clinicians ask clinical questions to ChatGPT; however, it is unclear if most clinicians are aware of detailed prompt functionality and know how to effectively use it in their routine practice.

The number of responses deemed useful by Koh and colleagues was higher than in our study. Of note, they had 2 investigators who were ID physicians evaluate the responses, as opposed to 5 ID pharmacists in our study. There is also likely some difference in evaluation standards among health care professionals despite using the same definitions. The generated questions were clinical ID pharmacotherapy questions that ID pharmacists receive and are perhaps best equipped to answer on the basis of their education, training, and clinical experience. ID physicians rating these responses as useful may have some degree of rater bias with determining that the responses are useful, when there are indeed potential limitations with the GPT-4o responses. In contrast, ID pharmacists would likely be much more satisfied with ChatGPT responses to ID diagnostic questions than ID physicians would be.

While we do not have the specific rating scores for each response, we did evaluate some of the GPT-4o responses for accuracy in our interpretation of useful responses. The response to question 83 suggests using an alternative antibiotic to cefazolin for surgical site prophylaxis in a patient with anaphylaxis to penicillin despite cefazolin being able to be used in this scenario [7]; therefore, this would not be rated as useful. Furthermore, some responses still hallucinate. For example, the response to question 99 states that ampicillin susceptibility does not imply meropenem susceptibility in *Enterococcus faecalis*, which is appropriate and useful; however, the response further states that enterococci are inherently resistant to carbapenems due to a lack of target affinity, which is incorrect and not useful since imipenem-cilastatin does indeed have activity against *E faecalis*, with ampicillin serving as a surrogate for susceptibility [8]. There are also some issues with the sources cited in the responses. For example, the response to question 27 cited a source of “Infectious Diseases Society of America Epidural Abscess Treatment Recommendations,” yet such guidelines do not currently exist. While these are only a couple of examples that raise concern, these help to support that the useful rating of 93% may be overstated.

The rapidly changing nature of the artificial intelligence field and the challenges of keeping up with peer-reviewed publication enterprise make evaluations of artificial intelligence on clinical practice challenging. Perhaps a future opportunity could include presenting models with more real-world ID cases that focus on balancing risks and benefits to see how ChatGPT compares with human clinical judgment and priorities. Nonetheless, we agree with Koh and colleagues that while GPT-4o improved some of the shortcomings identified with GPT-3.5, ChatGPT still cannot replace a human health care professional's judgment and management in clinical practice.

## Notes

***Ethics statement*.** This letter to the editor does not directly involve any patients or confidential information and therefore did not require any ethics review or approval for its conduct.
